# Unraveling Cannabidiol’s Bidirectional Regulation of Melatonin Pharmacokinetics via PEPT1/CYP1A2: Mechanistic Insights and Quantitative Projections

**DOI:** 10.3390/ph19010080

**Published:** 2025-12-30

**Authors:** Bohong Zheng, Mengran Wang, Qiannan Zhang, Cong Li, Lingchao Wang, Wenpeng Zhang, Chunyan Liu, Xiaomei Zhuang

**Affiliations:** 1 Academy of Military Medical Sciences, Beijing 100850, China; zhengbohong2000@163.com (B.Z.); 18112040512@163.com (M.W.); zqn1217yolo@163.com (Q.Z.); seancong1005@163.com (C.L.); 13426199839@163.com (L.W.); wpzhang@bmi.ac.cn (W.Z.); 2 State Key Laboratory of National Security Specially Needed Medicines, Beijing 100039, China; 3 School of Pharmacy, North China University of Science and Technology, Tangshan 063210, China; chunyanliu@ncst.edu.cn

**Keywords:** melatonin, cannabidiol, CYP1A2, PEPT1, drug-drug interaction

## Abstract

**Background:** Chronic insomnia is associated with elevated cardiovascular disease risk, and current therapeutic options for this condition remain inadequate. Melatonin (MT) combined with cannabidiol (CBD) may exert synergistic effects on improving sleep; the underlying pharmacological drug–drug interactions (DDI) and interspecies differences in their combined actions remain unknown. **Purpose:** This study aimed to evaluate the pharmacokinetic characteristics of combined drug formulations by utilizing DDI-based approaches so as to underpin the efficacy and safety of the formulation. **Methods:** Overexpressing hPEPT1 in MDCK cells, multiple species liver microsomes, equilibrium dialysis, and a static DDI model were employed to assess CBD’s effects on MT’s cellular uptake, inhibitory effect, enzymatic phenotype, protein binding, and human AUC changes. **Results:** CBD significantly increased MT exposure in dogs but caused dose-dependent biphasic changes in rats. MT negligibly affected CBD PK. In vitro, CBD inhibited MT metabolism with species differences: potent competitive inhibition in dogs (IC_50_ = 3.42 ± 1.30 μM), weaker inhibition in rats/humans (IC_50_ = 13.54 ± 1.15/16.47 ± 4.23 μM). CBD also demonstrated mechanism-based inhibition (K_I_ = 25.63 μM, K_inact_ = 0.063 min^−1^) against human CYP1A2-mediated MT metabolism. Acidic conditions revealed that CBD inhibited PEPT1-mediated MT uptake. CBD exhibits high and MT moderate protein binding. Static model predictions aligned with in vivo dog/rat data project a worst-case human MT AUC increase up to 12-fold. **Conclusions:** This study identifies the critical role of PEPT1 in MT absorption and elucidates the dual mechanisms of CBD; namely, absorption inhibition and metabolic delay in regulating MT pharmacokinetics, which exhibits interspecies differences.

## 1. Introduction

Chronic insomnia has emerged as a growing public health challenge worldwide [1]. Its pathological features are not only reflected in core symptoms such as sleep onset latency and sleep maintenance disturbances, but they also exhibit a significant association with elevated cardiovascular disease risk via intricate pathophysiological pathways [2,3]. Epidemiological evidence consistently indicates that patients with insomnia and objectively reduced sleep duration show a significantly higher incidence of hypertension compared with the general population, underscoring the important role of sleep quality in cardiovascular risk management [4,5]. Current clinical management of insomnia relies primarily on two approaches: cognitive behavioral therapy for insomnia (CBT-I) and pharmacotherapy [6]. However, the widespread adoption of CBT-I is limited by practical constraints, including the need for specialized training and challenges in maintaining long-term patient compliance [7]. In contrast, existing first-line therapeutic agents [8], including benzodiazepine receptor agonists and dual orexin antagonists, are associated with drawbacks such as tolerance, daytime sedation, and potential cognitive impairment with prolonged use, which may restrict their clinical utility [9]. Notably, melatonin (MT) and cannabinoids (CBD), owing to their unique neuroendocrine regulatory mechanisms and sleep-wake cycle regulation properties, have offered a novel research paradigm [10] for the development of innovative therapeutic agents for insomnia. These compounds have emerged as research frontiers in sleep medicine and pharmacology in recent years.

As an indoleamine hormone synthesized and secreted by the pineal gland, MT regulates circadian rhythms by activating MT1/MT2 receptors [11]. Exogenous supplementation of MT has been confirmed to exert a dose-dependent effect in shortening sleep latency. Further pharmacological studies have revealed multiple biological activities of MT [12], including free radical scavenging, immune response modulation, and inhibition of inflammatory factor release. Cannabidiol [13], a non-psychoactive and non-addictive component in *Cannabis* plants, modulates the 5-hydroxytryptamine 1A receptor (5-HT1A) signaling pathway by regulating the endocannabinoid system (ECS) [14]. It has demonstrated significant anxiolytic [15] and sleep-promoting effects [16] in animal models. Recent studies have shown that, compared with single-drug administration, the combined administration of CBD and MT in insomnia models of rats and mice significantly prolongs slow-wave sleep duration (*p* < 0.05) [17]. This observation suggests the potential existence of a synergistic effect or pharmacokinetic (PK) interaction between the two compounds [18].

It is known that melatonin (MT) is primarily metabolized via CYP1A1/1A2 to form 6-hydroxymelatonin (6-HMT) [19,20], whereas CBD is metabolized via CYP2C9/2C19 to 7-hydroxy-CBD [21,22,23], with no competitive interaction observed between the two compounds in their respective metabolic pathways. However, in vitro studies have confirmed that CBD exerts a concentration-dependent inhibitory effect on CYP1A1/2 enzymes [24], and no direct evidence currently exists regarding whether CBD modulates MT’s clearance rate. Given the observed benefits of co-administration in improving sleep in preclinical studies, the following issues require in-depth investigation: (1) Whether CBD modulates MT’s bioavailability by inhibiting CYP1A-mediated first-pass metabolism of MT; (2) Whether the synergistic effect of MT-CBD co-administration arises from PK interactions or is primarily driven by pharmacodynamic synergy; (3) Whether interspecific differences in metabolic enzyme activity lead to variations in the nature and mechanism of their interaction during co-administration.

To address the aforementioned research questions, the following steps will be implemented: first, systematically characterize the ADME (Absorption, Distribution, Metabolism, and Excretion) properties of CBD-MT co-administration through in vivo animal experiments; second, quantitatively analyze the regulatory mechanism of CBD on the intestinal absorption and metabolic conversion of MT using MDCK/PEPT1/2 cell models and liver microsomes incubation systems; finally, establish a inhibition based static model to predict the magnitude of CBD-MT interation in humans. This study is expected to provide critical evidence to support the clinical translation of the combined therapeutic regimen involving the natural compounds CBD and MT.

## 2. Results and Discussion

### 2.1. Pharmacokinetic Results in Beagles

**Figure 1:**
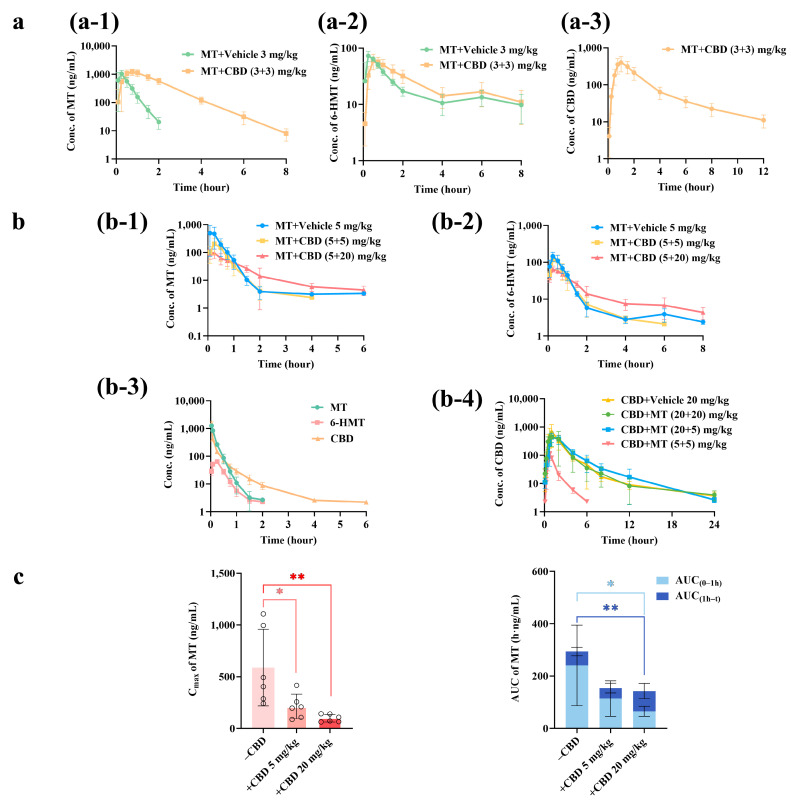
Pharmacokinetic profiles of MT and CBD in beagles and rats and the relationships between the exposure of CBD and MT ($\bar{x}$ ± s, *n* = 6). (**a**) Plasma concentration–time curves of MT, 6-HMT, and CBD after oral administration in beagles. Plasma concentration–time curves of MT after co-administration of MT and CBD in dogs (**a-1**). Plasma concentration–time curves of 6-HMT after co-administration of MT and CBD in dogs (**a-2**). Plasma concentration-time curves of CBD after co-administration of MT and CBD in dogs (**a-3**). (**b**) Plasma concentration–time curves of MT after oral co-administration of MT and different doses of CBD in rats (**b-1**). Plasma concentration–time curves of 6-HMT after co-administration of MT and different doses of CBD in rats (**b-2**). Plasma concentration–time curves of CBD after oral co-administration of MT and CBD in rats (**b-3**). Plasma concentration–time curves of MT, 6-HMT, and CBD in rats after *i.v.* administration of MT and CBD (**b-4**). (**c**) Comparison of MT exposure (C_max_ and AUC) after co-administration of MT and different doses of CBD in rats. *: *p* < 0.05, there was a statistical difference, **: *p* < 0.01.

The plasma concentration–time curves of MT, 6-HMT, and CBD in Beagles of different groups are shown in Figure 1a, with the corresponding pharmacokinetic parameters listed in Table 1. After oral administration of MT alone (3 mg/kg), the plasma concentration of MT reached the peak rapidly, followed by rapid metabolic elimination. When co-administered with CBD, the C_max_ of MT increased, while T_max_, MRT, and t_1/2_ were prolonged; the AUC_(0–t)_ of MT was 4.2-fold that of the MT alone group. Although the exposure of 6-HMT showed no significant difference, the ratio of AUC_(0–t),6-HMT_ to AUC_(0–t),MT_ decreased to 26% (from 0.3 to 0.08).

### 2.2. Pharmacokinetic Characteristics of MT in Rats

The concentration–time curves of MT and 6-HMT after intravenous and oral administration of MT and CBD to SD rats are shown in Figure 1b. The results of correlation analysis between C_max_ and AUC of MT and CBD doses are presented in Figure 1c, and the pharmacokinetic parameters of MT and 6-HMT are listed in Table 2, respectively. Following intravenous injection of MT + CBD (1 + 1) mg/kg in rats, the apparent volume of distribution of MT (1.16 L/kg) was larger than the plasma volume of rats (0.0312 L/kg), and the clearance rate (3.88 L/h/kg) slightly exceeded the hepatic blood flow in rats (3.33 L/h/kg). When rats were given oral administration of 5 mg/kg MT alone, it also exhibited the characteristics of rapid metabolic clearance after rapid absorption, with a short residence time in the body (0.67 h) and an oral bioavailability of 20.5% for MT. With the increase in the dose of co-administered oral CBD, the C_max_ of MT gradually decreased, and the exposure in the absorption phase, AUC_(0–1h)_, showed a decreasing trend. After co-administration with 20 mg/kg CBD, the behavior of the MT drug–time curve changed significantly, with a slower elimination rate, a significant prolongation of t_1/2_, and an increase in the exposure in the metabolic elimination phase, AUC_(1h–t)_. Although the overall exposure AUC_(0–t)_ decreased, the MRT was prolonged. After co-administration of different doses of CBD with MT, the C_max_ of the metabolite 6-HMT and the exposure in the absorption phase, AUC_(0–1h)_, also decreased, which was consistent with the changes in the parent MT. When co-administered with 20 mg/kg CBD, the exposure in the metabolic elimination phase, AUC_(1h–t)_, of 6-HMT increased significantly, while the overall exposure showed no obvious change, and the MRT was prolonged.

### 2.3. Pharmacokinetic Characteristics of CBD in Rats

The pharmacokinetic parameters of CBD after oral administration of MT + CBD (5 + 20) mg/kg, CBD 20 mg/kg, and MT + CBD (20 + 20) mg/kg to SD rats are shown in Table 3. When CBD was co-administered with different doses of MT, there were no statistically significant differences in its pharmacokinetic parameters, indicating that MT does not produce obvious pharmacokinetic-based interactions with CBD.

### 2.4. Effect of CBD on the Pharmacokinetics of MT in Rats After Co-Administration with 1-Aminobenzotriazole (ABT)

**Figure 2:**
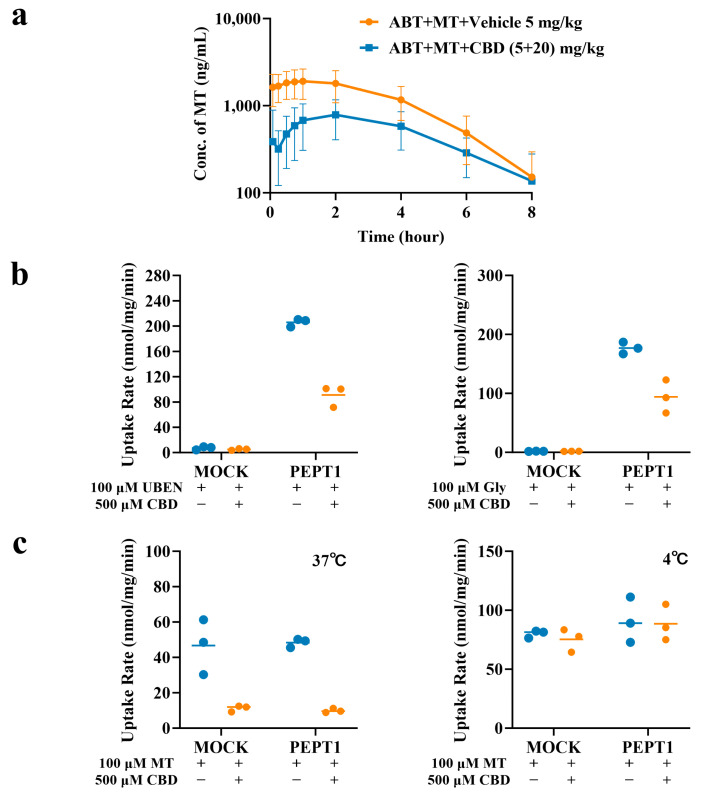
Inhibition of PEPT1 Uptake Function by CBD. (**a**) Plasma concentration–time curves of MT after pre-administration of ABT and co-administration of MT and CBD in rats ($\bar{x}$ ± s, *n* = 6). (**b**) Inhibition of CBD on UBEN and Gly uptake in MDCK/hPEPT1 cell model ($\bar{x}$ ± s, *n* = 3). (**c**) Inhibition of MT uptake by CBD in MDCK/hPEPT1 cell model ($\bar{x}$ ± s, *n* = 3).

After chemical knockout of CYP function by co-administration with ABT, the plasma concentration–time curves of MT in rats after oral administration of MT 5 mg/kg alone and MT + CBD (5 + 20) mg/kg are shown in Figure 2a, and the pharmacokinetic parameters of MT and CBD are listed in Table 4. In SD rats pretreated with ABT, the AUC of MT after oral administration of MT 5 mg/kg alone was approximately 33.1-fold that of rats not co-administered with ABT. After co-administration with ABT followed by MT + CBD (5 + 20) mg/kg, the AUC of MT also reached approximately 31.8-fold that of rats not treated with ABT. Furthermore, only trace amounts of 6-HMT were detected in the plasma of the two groups of rats after ABT co-administration (plasma concentrations at all sampling time points were below the lower limit of quantification of 2 ng/mL), indicating that chemical knockout of rat CYP enzymes by ABT almost completely inhibited the metabolism of MT, confirming the successful establishment of the model. Comparison of the plasma concentration–time curves and pharmacokinetic parameters of MT in ABT-model rats after combined oral administration of MT + CBD (5 + 20) mg/kg with those in ABT-model rats after oral administration of 5 mg/kg MT alone showed a clear decrease in C_max_ and AUC_(0–t)_ of MT (both by approximately 43%), along with a prolonged half life and delayed time to peak concentration of MT. These results suggest that when the function of CYP enzymes is inhibited, CBD mainly exerts an effect of suppressing the active intestinal absorption of MT. Simultaneous determination of plasma CBD concentrations in ABT-model rats after oral administration of MT + CBD (5 + 20) mg/kg showed that, compared with the control group, ABT also affected the metabolism of CBD, with its AUC_(0–t)_ being approximately 1.68-fold that of the control group. This indicates that the metabolism of CBD was also inhibited, but the extent of the effect was significantly weaker than that on MT.

### 2.5. Inhibition of PEPT1 Uptake Function by CBD

The results of CBD’s inhibition on the uptake of PEPT transporter positive substrates ubenimex (UBEN) and Gly are shown in Figure 2b. The uptake rates of UBEN and Gly in this model were 28.6 and 93.2, respectively, confirming the good performance of this uptake system. The inhibition rates of CBD on the uptake of UBEN and Gly via PEPT1 were 55.8% and 46.7%, respectively, suggesting that CBD is an effective inhibitor of PEPT1.

The results of CBD’s inhibition on MT in the MDCK/PEPT1 cell model are shown in Figure 2c. Under incubation conditions of 4 °C and 37 °C, there was no difference in the uptake rate of MT between MOCK cells and transfected cells. After co-incubation with CBD, the uptake rate of MT in the 4 °C group remained unchanged (with an uptake inhibition rate of 2.7%), while at 37 °C, the uptake inhibition rate of MT by CBD was 79.6%. These results indicate that the inhibition of PEPT1-mediated MT uptake by CBD is dependent on the 37 °C condition, and CBD can inhibit the intestinal PEPT1-mediated uptake of MT, which is consistent with the in vivo results.

### 2.6. Protein Binding and Blood-to-Plasma Ratio of CBD and MT

To calibrate and convert effective drug concentrations between in vitro and in vivo experiments, the blood-to-plasma ratio (R_b/p_) and protein binding rates of CBD and MT were determined across species. Results are summarized in Table 5.

CBD exhibited species-dependent R_b/p_ values: In beagle dogs, the R_b/p_ was 1.07, indicating that plasma concentrations reliably represent whole-blood concentrations. Conversely, in rats and humans, R_b/p_ ranged from 0.52 to 0.63, suggesting uneven distribution in whole blood; thus, plasma concentrations cannot accurately reflect blood concentrations. In contrast, MT concentrations in whole blood were essentially equivalent to those in plasma.

CBD demonstrated high protein binding (>99%) with both hepatic microsomes and plasma proteins across species, showing no significant interspecies differences. MT, however, displayed markedly lower protein binding than CBD and is classified as a low-to-moderate plasma protein-bound drug.

### 2.7. CYP Enzyme Metabolic Phenotyping of MT

**Figure 3:**
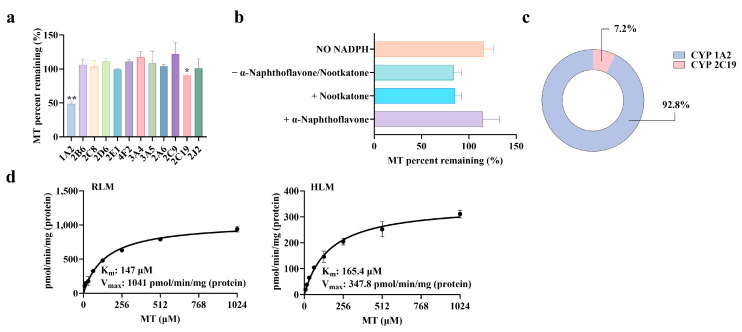
Characteristics of MT Metabolism Mediated by CYP Enzymes. (**a**) Metabolic phenotype of MT (recombinant enzyme method) ($\bar{x}$ ± s, *n* = 3). (**b**) Metabolic phenotype of MT (inhibitor method) ($\bar{x}$ ± s, *n* = 3). (**c**) Metabolic contribution fraction of CYP isoenzyme to MT. (**d**) Enzymatic kinetics of MT 6-hydroxylation reaction ($\bar{x}$ ± s, *n* = 3). (*: *p* < 0.05, there was a statistical difference, **: *p* < 0.01, there was a significant statistical difference, compared with the 0-hour time point).

The positive control drugs were normally metabolized in the corresponding recombinant human isozymes, indicating that the recombinant human CYP isozymes in the incubation system had robust activity and were suitable for the study on metabolic enzyme phenotyping of MT. MT remained stable in all recombinant human isozymes without NADPH. The residual percentage of MT parent drug in the groups with NADPH is shown in Figure 3a. The results of the inhibitor-treated groups are shown in Figure 3b, and the metabolic contributions of isozymes to MT are shown in Figure 3c. The results showed that MT was metabolically transformed via CYP1A2 and CYP2C19, which was consistent with the results reported in the literature [25,26]. In this study, the normalized method was applied for the first time to quantitatively calculate the metabolic contribution rate of CYP1A2 to MT, which was 92.8%, followed by CYP2C19 with a contribution rate of 7.2%.

### 2.8. Enzyme Kinetics of MT 6-Hydroxylation

The results of enzyme kinetic experiments on MT 6-hydroxylation in rat and human liver microsomes are shown in Figure 3d. The nonlinear pharmacokinetic process was characterized by the kinetic parameters K_m_ and V_max_, which are characteristic of enzyme kinetics. There was no significant difference in K_m_ values of MT between rat and human liver microsomes (147 vs. 165 μM). However, the V_max_ in rats was higher than that in humans (1041 vs. 347.8 pmol/min/mg protein), suggesting that the clearance rate of MT in rats is higher than that in humans.

### 2.9. Lack of Effect of MT on In Vitro Metabolism of CBD

**Figure 4:**
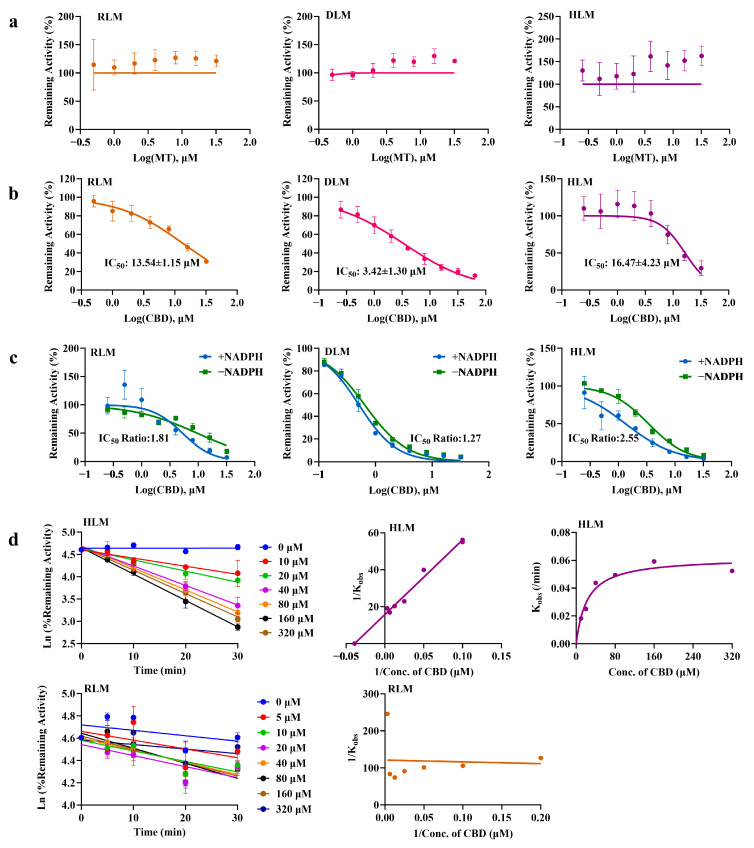
Drug Metabolism-Based Interaction Between CBD and MT. (**a**) Competitive inhibition of CBD by MT in liver microsomes ($\bar{x}$ ± s, *n* = 3); (**b**) Competitive inhibition of MT by CBD in liver microsomes ($\bar{x}$ ± s, *n* = 3); (**c**) Time-dependent inhibition of CBD on MT ($\bar{x}$ ± s, *n* = 3); (**d**) Irreversible inhibition of MT by CBD ($\bar{x}$ ± s, *n* = 3).

As shown in Figure 4a, in the incubation systems of liver microsomes from different species, the concentration of CBD did not decrease significantly with changes in MT concentration, indicating that MT does not exert competitive inhibition on CBD.

### 2.10. Competitive Inhibition of CBD on MT 6-Hydroxylation

The results of competitive inhibition of CYP enzymes involved in melatonin 6-hydroxylation by CBD are shown in Figure 4b. The results indicate that there is a significant species difference in the inhibitory effect of CBD on MT. Specifically, the inhibitory effect in beagle dog liver microsomes is significantly stronger than that in liver microsomes of rats and humans.

### 2.11. Time-Dependent Inhibition of CBD on MT 6-Hydroxylation

The results of time-dependent inhibition of CBD on MT 6-hydroxylation in liver microsomes, determined by the IC_50_ shift method, are shown in Figure 4c. After pre-incubation of human liver microsomes with CBD and NADPH, the IC_50_ shift value of CBD on MT exceeded 2, suggesting that the metabolites of CBD in human liver microsomes may exert inhibitory effects on MT 6-hydroxylation, indicating a potential time-dependent inhibition. In contrast, the IC_50_ shift values in other liver microsomes were all less than 2, indicating that the time-dependent inhibition of CBD on MT exhibits species differences.

### 2.12. Irreversible Inhibition of CBD on MT 6-Hydroxylation

Liver microsomes from two species with a relatively large degree of IC_50_ shift in the TDI experiment were selected for irreversible inhibition assays. Using enzyme inactivation reaction experiments, the K_I_ value of CBD for CYP enzymes mediating MT 6-hydroxylation in human liver microsomes was determined to be 25.63 μM, with a K_inact_ value of 0.063/min. The semi-logarithmic curve and double reciprocal curve are shown in Figure 4d. This irreversible inhibition mechanism suggests that CBD may persistently affect the activity of CYP1A2 through covalent modification, allosteric regulation, or other mechanisms [27]. In contrast, no irreversible inhibitory effect was observed in rat liver microsomes.

### 2.13. Prediction of CBD’s Impact on MT Exposure via Static Model

**Figure 5:**
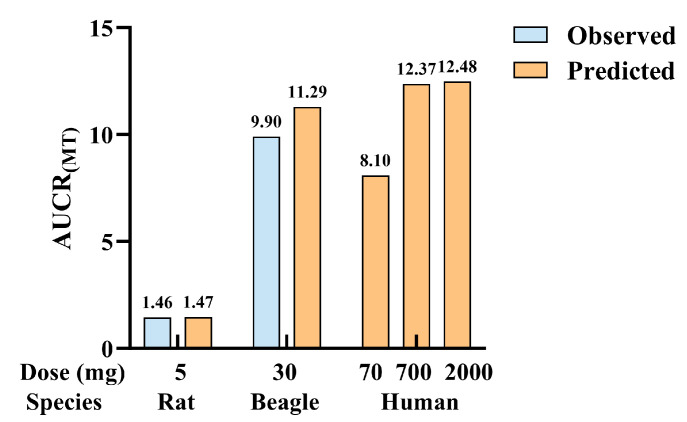
Prediction of the degree of inhibition of MT 6-hydroxylation by CBD of different species.

The static model approach [28,29] was employed to calculate the degree of CBD-mediated inhibition on MT metabolism across different species, with the predicted results presented in Figure 5. The findings demonstrated that, after precise calibration of the effective drug concentrations of CBD in in vitro and in vivo systems using the static model, the predicted inhibitory effects of CBD on MT metabolic clearance in rats and beagle dogs were consistent with the actual values. In vitro studies revealed that CBD exerts a mixed effect of competitive and mechanism-based inhibition on human CYP1A2-mediated MT metabolism. Based on reported in vivo concentrations of CBD following oral administration of 70–2000 mg in humans [30], the predicted inhibitory effect on MT metabolic clearance (expressed as AUCR) could reach 8.10–12.48. Collectively, the model predictions indicate that CBD exhibits high potential to inhibit MT 6-hydroxylation in humans. Notably, the above results only evaluate the changes in MT exposure attributed to CBD-mediated CYP inhibition, where the measured AUC values were calculated using data from the distributive-elimination phase. Since CBD also inhibits intestinal PEPT1, the actual in vivo outcomes are expected to be lower than the predicted results, and the in vivo concentration of MT is anticipated to be more stable.

## 3. Discussion

This study systematically investigates the pharmacokinetic interactions and underlying mechanisms of CBD and MT during co-administration. It is the first to demonstrate that CBD sustains more stable plasma concentrations of MT after oral dosing via a dual mechanism: concurrent inhibition of PEPT1-mediated active intestinal absorption of MT and CYP1A2-dependent hepatic metabolic degradation of MT. In contrast, MT has negligible impact on CBD’s pharmacokinetic profile.

First, in the pharmacokinetic studies in rats and beagles, MT displayed consistent profiles of rapid absorption and rapid elimination across both species-a feature that may contribute to MT’s ability to facilitate sleep initiation yet limit its efficacy in sustaining sleep. Notably, notable interspecies differences emerged in the effects of CBD on MT pharmacokinetics during co-administration: in rats, as CBD dose increased, the C_max_ of MT initially decreased in a dose-dependent manner, whereas exposure during the distribution and elimination phase increased with the CBD dose. Although MT’s total exposure was reduced (declining to 45% at the high CBD dose), plasma concentrations remained more stable overall, and the MRT was prolonged (increased to 1.98-fold at the high CBD dose). In contrast, in beagles, co-administration of CBD increased the C_max_ of MT (up to 1.3-fold at the high CBD dose) and markedly enhanced MT’s total exposure, with AUC increasing to 4.2-fold.

To investigate the mechanisms underlying CBD-induced alterations in MT pharmacokinetic properties and the associated interspecies differences, a series of in vitro experiments was conducted. First, in the metabolic enzyme phenotyping assay, we confirmed that CYP1A2 is the primary metabolic enzyme mediating MT 6-hydroxylation, with a quantified metabolic contribution ratio of 92.8%. In hepatic microsomal enzyme kinetic experiments, although the K_m_ value for the MT 6-hydroxylation was comparable between rat and human hepatic microsomes, the maximum reaction rate (V_max_) in rats was significantly higher than that in humans, which explains the rapid elimination profile of MT in rats. Second, the inhibitory effect of CBD on MT metabolism was compared in hepatic microsomes from different species. Although CBD has been confirmed to inhibit CYP1A2, its inhibitory effect on MT metabolism has not been previously reported. In beagle hepatic microsomes, CBD exerted a relatively strong competitive inhibitory effect on MT (IC_50_ = 3.36 μM), whereas its inhibitory effect was weaker in other species (13.54–36.45 μM). Further pre-incubation experiments revealed that the inhibitory effect of CBD on MT 6-hydroxylation exhibited time-dependent characteristics only in human hepatic microsomes, manifesting as mechanism-based irreversible inhibition (K_I_ = 25.63 μM, K_inact_ = 0.063/min), while no such effect was observed in other species. These interspecies differences in CBD-induced inhibition of MT metabolism, observed in rat and beagle dog hepatic microsomal experiments, can explain the discrepancy in their in vivo co-administration outcomes.

In rats, the complex alterations in MT pharmacokinetic profiles induced by CBD may involve regulation of MT intestinal absorption, in addition to CYP1A2 inhibition. Intestinal epithelial cells express a variety of transporters, primarily including uptake transporters that mediate efficient absorption of glucose, amino acids, and peptides, and efflux transporters that reduce the absorption of toxic substances. Given that CBD co-administration decreases MT absorption, it may inhibit MT’s active uptake. Considering the high expression of PEPT1 on the apical membrane of intestinal epithelial cells [31,32] and the amphiphilic properties of MT (which are similar to those of PEPT1 substrates), it was hypothesized that PEPT1 may serve as a key transporter mediating the intestinal absorption of MT. To verify this mechanism, the effect of CBD on MT uptake into cells was investigated in MDCK cells expressing PEPT1. PEPT1 is a transporter in intestinal epithelial cells that actively uptakes small peptides and certain drugs through an H^+^-coupled mechanism [33]. Under acidic conditions, CBD exhibited an inhibitory effect on the active uptake of classical substrates (UBEN and Gly) via PEPT1 [33,34], indicating that CBD is a PEPT1 inhibitor. Further comparisons of CBD’s effects on MT uptake under 4 °C (inhibiting active transport) and 37 °C (permitting active transport) conditions confirmed that CBD can suppress PEPT1-mediated active uptake of MT. Literature reports indicate that PEPT1 transporters are present in the pineal gland of rats, with expression exhibiting distinct circadian rhythms, suggesting that endogenous melatonin is a substrate of PEPT [35,36]. In MOCK-MDCK cells, CBD also significantly reduced the uptake rate of MT. This phenomenon is presumably attributed to the fact that MDCK cells (derived from canine renal epithelial cells) may endogenously express PEPT2 (a transporter also involved in MT active transport), and CBD may exert inhibitory effects on PEPT2 as well. The specific mechanism underlying this observation requires further investigation. To further validate that CBD inhibits not only CYP1A2-mediated MT metabolism but also PEPT1-dependent MT uptake, a study was conducted in rats to evaluate CBD’s effect on MT following chemical inhibition of CYP enzymes. Post intragastric administration of ABT to rats, in vivo MT exposure increased by 33-fold when MT was administered alone, indicating that ABT potently inhibited CYP1A2 activity. Under this condition, co-administration of CBD with MT resulted in a downward shift of the overall plasma concentration–time curve, with C_max_ and AUC reduced by 2.3-fold and 2.29-fold, respectively. These results suggest that in the absence of CYP enzyme-mediated metabolic interference, CBD primarily decreases MT absorption rate and extent by inhibiting the intestinal PEPT1 transporter. Notably, studies have reported significant differences in the expression levels and functional activities of intestinal drug transporters between rodents and canines [37]—a factor that may explain the interspecies differences in CBD’s regulatory effect on MT absorption.

Differences in the pharmacokinetic behaviors of CBD and MT co-administration between rats and beagle dogs, when integrated with in vitro metabolic enzyme inhibition data, reveal significant species-specific differences in the pharmacological effects of their co-administration. Consequently, pharmacokinetic-based DDI data derived from animal studies cannot be reliably extrapolated to humans. Furthermore, efficacy findings from animal models may also fail to predict outcomes in humans. Furthermore, given the inherent coupling of pharmacokinetics and pharmacodynamics (PK-PD), efficacy observations obtained from animal models may also be unable to accurately predict clinical outcomes in humans, particularly considering the species-dependent variations in both drug disposition (e.g., transporter/enzyme activity) and target-mediated responses identified in this study.

To preliminarily assess the potential in vivo DDI between CBD and MT in humans and its clinical relevance, quantitative in vitro–in vivo extrapolation (IVIVE) was performed using a static model. To improve the accuracy of IVIVE predictions, we determined cross-species parameters of CBD and MT, including R_b/p_ and f_up_, to calculate the free CBD concentration at the liver inlet. Human-derived data were acquired from the literature [30]. Given that CBD exerts a mixed mode of competitive and noncompetitive inhibition toward MT metabolism in human liver microsomes, both inhibition modes were integrated into the static prediction model. Considering the weak CYP1A2-mediated inhibitory effect of CBD on MT in rats, as well as the confounding effect of intestinal PEPT1 inhibition during the absorptive phase, we only quantified changes in MT exposure during the distribution–elimination phase. Static model predictions revealed that the inhibitory effect of CBD on MT metabolism in humans (expressed as AUCR) could reach 12.48-fold, a magnitude of effect with potential clinical relevance. Noting that the therapeutic plasma concentration of CBD in humans typically spans from 1 to 10 μM, our findings suggest that dosage adjustments of MT may provide a reference for dosage adjustment considerations in potential future clinical trials of CBD-MT combination therapy.

Despite the valuable findings of this study, several limitations should be acknowledged: First, the CBD doses employed in animal experiments may not fully encompass the clinically relevant concentration range, which could restrict the translational relevance of the observed in vivo DDI patterns. Second, the specific contribution of the PEPT1 transporter to MT’s intestinal absorptive process in humans, as well as its associated quantitative parameters (e.g., transport efficiency, fraction absorbed via PEPT1), has not been directly verified, limiting the accuracy of extrapolating intestinal absorption regulation mechanisms to the human population. Additionally, this study only investigated the impact of CBD on the pharmacokinetics profiles of MT; given that CBD itself exhibits sleep-regulating properties, the direct influence of their co-administration on MT’s pharmacodynamic effects (e.g., sleep initiation/sustainment efficacy) remains to be further elucidated.

Future research could be expanded in the following directions: Using PEPT1 gene knockout animal models to directly validate the role of this transporter in MT’s absorption, thereby quantifying its contribution to overall MT bioavailability. Exploring the regulatory effect of long-term CBD administration on the pharmacokinetics of MT, as chronic exposure may induce adaptive changes in metabolic enzymes (e.g., CYP1A2) or transporters (e.g., PEPT1) that differ from acute co-administration. Conducting clinical studies to evaluate the pharmacokinetic characteristics of the CBD-MT combination regimen in humans, with simultaneous monitoring of PD endpoints (e.g., sleep latency, slow-wave sleep duration), was done to establish PK-PD correlations and guide rational dosage regimens.

In summary, this study offers both distinct theoretical insights and practical implications. At the theoretical level, it is the first to systematically elucidate the key role of the PEPT1 transporter in MT’s intestinal absorption while defining the scientific mechanism by which CBD regulates MT pharmacokinetics via dual pathways: concurrent inhibition of PEPT1-mediated intestinal absorption and CYP1A2-dependent metabolic clearance of MT. This finding addresses a critical gap in understanding regulatory crosstalk in MT disposition by CBD, providing novel perspectives on pharmacokinetic-based drug–drug interactions between CBD and MT. Practically, the results establish a key pharmacokinetic rationale for the development of CBD-MT combination formulations. Given MT’s inherent PK limitations (e.g., rapid absorption and elimination), the study suggests that optimizing the CBD-MT compatibility ratio may mitigate such defects—for instance, by prolonging MT’s mean residence time (MRT) and stabilizing its plasma concentrations, thereby laying a foundation for improving the clinical efficacy and safety of the combination regimen.

## 4. Materials and Methods

### 4.1. Materials

The FRESCO21 centrifuge, multifunctional microplate reader, and Pierce^®^ BCA Protein Quantification Kit were purchased from Thermo Fisher Scientific (Waltham, MA, USA). The T-214 electronic analytical balance was purchased from Denver Instrument Co., Ltd. (Denver, CO, USA). The LC-MS/MS-8060 triple quadrupole liquid chromatography-tandem mass spectrometer was from Shimadzu Corporation (Tokyo, Japan). The homogenizer and controlled constant temperature shaking incubator were purchased from IKA-Werke GmbH & Co. KG (Staufen, Germany). The microscope was purchased from Nikon Corporation (Tokyo, Japan). The CO_2_ incubator was purchased from Shanghai Puhexi Health Medical Devices Co., Ltd. (Shanghai, China).

Melatonin (≥98%) and Hank’s buffer were purchased from Beijing Solarbio Science & Technology Co., Ltd. (Beijing, China). Melatonin-D4 and MES buffer were obtained from Shanghai Macklin Biochemical Technology Co., Ltd. (Shanghai, China). 6-Hydroxymelatonin (≥98%) was purchased from Shanghai Rhawn Chemical Technology Co., Ltd. (Shanghai, China). Cannabidiol (≥98%) was acquired from Wuhan Zhongbiao Technology Co., Ltd. (Wuhan, China). Ubenimex was purchased from Shanghai Jizhi Biochemical Technology Co., Ltd. (Shanghai, China). Gly-Sar and castor oil were obtained from Shanghai Yuanye Bio-Technology Co., Ltd. (Shanghai, China). 1-Aminobenzotriazole was purchased from Beijing Wokai Biotechnology Co., Ltd. (Beijing, China). DMSO, bovine serum albumin, and PBS buffer were obtained from Sigma-Aldrich (St. Louis, MO, USA). Formic acid was purchased from Beijing J&K Scientific Co., Ltd. (Beijing, China). Ammonium acetate was acquired from Sinopharm Group Co., Ltd. (Shanghai, China). Purified water was purchased from Hangzhou Wahaha Group Co., Ltd. (Hangzhou, China). Penicillin-streptomycin, DMEM medium, and fetal bovine serum were obtained from Thermo Fisher Scientific (Waltham, MA, USA). Liver microsomes were purchased from XenoTech (Lenexa, KS, USA). Recombinant CYP enzymes were acquired from Bioreclamation-IVT Holdings (Wilmington, DE, USA). NADPH was purchased from Beijing Dingguo Changsheng Biotechnology Co., Ltd. (Beijing, China). α-Naphthoflavone and nootkatone were obtained from Tokyo Chemical Industry Co., Ltd. (Tokyo, Japan). Phenacetin, bupropion, and coumarin were purchased from Selleck Chemicals (Houston, TX, USA). Amodiaquine, S-mephenytoin, and astemizole were acquired from Toronto Research Chemicals (Toronto, ON, Canada). Dextromethorphan and ketoconazole were purchased from USP (Rockville, MD, USA).

The MDCK and MOCK cell lines stably overexpressing PEPT1 were transfected by Hanheng Biotechnology Co., Ltd. (Shanghai, China). SPF-grade Sprague-Dawley (SD) rats (weighing 200–220 g) were obtained from Beijing Vital River Laboratory Animal Technology Co., Ltd. (Beijing, China); beagles were sourced from Beijing Mars Biotechnology Co., Ltd. (Beijing, China). The experimental protocol was approved by the Animal Care and Use Committee of Academy of Military Medical Sciences (IACUC-DWZX-2024-505).

### 4.2. Pharmacokinetics of CBD and MT in Beagle Dogs

Twelve male healthy adult beagles (weighing 10 ± 2 kg) were randomly divided into two groups. They were given a single intragastric administration of 3 mg/kg MT alone and a combination of MT and CBD at (3 + 3) mg/kg [17], respectively. The drugs were dissolved in a physiological saline solution containing 5% castor oil, with an administration volume of 2 mL/kg. Blood samples were collected at the time points of pre-administration and 0.083, 0.25, 0.5, 0.75, 1, 1.5, 2, 4, 6, 8, and 12 h post-administration. Approximately 100 μL of venous blood were collected into heparin sodium anticoagulant tubes and placed on ice. Within 1 h, the whole blood samples were centrifuged at 2500× *g* for 10 min at 4 °C, and the plasma was collected and stored in a −40 °C refrigerator for subsequent detection. The established LC-MS/MS method was used for quantitative analysis of MT, 6-HMT, and CBD in samples [38].

### 4.3. Pharmacokinetics of CBD and MT in Rats

Twenty-four male Sprague-Dawley (SD) rats (weighing 200 ± 20 g) were randomly divided into four groups, which were administered a single dose as follows: MT 1 mg/kg via intravenous injection, MT 5 mg/kg, MT + CBD (5 + 5) mg/kg, and MT + CBD (5 + 20) mg/kg via intragastric administration [17]. The drugs were dissolved in the same manner as described previously, with an administration volume of 5 mL/kg for all groups. Blood samples were collected at the time points of pre-administration and 0.033 (only for the intravenous administration group), 0.083, 0.25, 0.5, 0.75, 1, 1.5, 2, 4, 6, 8, and 12 h post-administration. Approximately 50 μL of venous blood were collected into heparin sodium anticoagulant tubes and placed on ice. Within 1 h, the whole blood samples were centrifuged at 2500× *g* for 10 min at 4 °C, and the plasma was harvested and stored in a −40 °C refrigerator for subsequent analysis.

### 4.4. Effect of CBD on the Pharmacokinetics of MT After Co-Administration with ABT

Twelve SD rats were randomly divided into two groups. Both groups were first given an intragastric administration of 1-aminobenzotriazole (ABT) at a dose of 200 mg/kg. Two hours later, they were administered an intragastric dose of MT 5 mg/kg and MT + CBD (5 + 20) mg/kg, respectively. ABT was dissolved in physiological saline, while MT and CBD were dissolved in physiological saline containing 5% castor oil. The administration volume for all groups was 5 mL/kg. Blood samples were collected at the time points before MT administration and at 0.083, 0.25, 0.5, 0.75, 1, 2, 4, 6, and 8 h after MT administration. Approximately 50 μL of venous blood were collected into heparin sodium anticoagulant tubes and placed on ice. The sample processing and storage methods were the same as described previously.

### 4.5. Inhibition of PEPT1-Mediated MT Uptake by CBD

MDCK-PEPT1 cells were seeded into 24-well plates at a density of approximately 5 × 10^5^ cells per well. When the cell confluency reached 60–70%, the culture medium was aspirated, and the cells were rinsed twice with HBSS pre-warmed to 37 °C. After removing the liquid in the wells, each well was filled with MES buffer (pH: 6.3) or MES buffer containing CBD, followed by pre-incubation at 37 °C for 30 min. The buffer was then aspirated, and MES buffer containing positive substrates (100 μM UBEN or 100 μM Gly) [34,39], MES buffer containing 100 μM MT, and MES buffer containing CBD (100 μM UBEN + 500 μM CBD, 100 μM Gly + 500 μM CBD, 100 μM MT + 500 μM CBD) were added, respectively. After incubation at 37 °C for 10 min, the buffer was aspirated, and ice-cold HBSS was immediately added to terminate the reaction, followed by rinsing three times. After drying the residual liquid, 200 μL of pure water were added to each well, and the cells were lysed by repeated freezing–thawing in liquid nitrogen three times. The cell lysates were collected, and the protein content was determined by the BCA method. After protein precipitation, the concentrations of UBEN, Gly, and MT were detected by LC-MS/MS. Meanwhile, a group of experiments for MT uptake under 4 °C conditions was designed, with other conditions consistent with those of the 37 °C incubation group.

### 4.6. Determination of Whole Blood/Plasma Ratio and Protein Binding

Fresh whole blood from different species (rat, beagle dog, and human) was pre-incubated at 37 °C for 5 min, then spiked with working solutions of CBD or MT to a final concentration of 1 μM (with organic solvent content less than 0.1%). After incubation for 20 min, a portion of the whole blood was centrifuged at 2500× *g* for 10 min at 4 °C to separate plasma; another portion of the whole blood was subjected to three cycles of freeze–thawing and ultrasonicated for 10 min to disrupt cells. Equal volumes of plasma and whole blood samples were added to the whole blood and plasma specimens, respectively, followed by protein precipitation with acetonitrile containing an internal standard (IS). A rapid equilibrium dialysis (RED) device was used to determine the protein binding rates of CBD and MT with liver microsomes from different species, as well as rat plasma, beagle dog plasma, and human plasma. The RED inserts were placed in the matching incubation plate: 200 µL of drug-containing matrix were added to the sample chamber, and 400 µL of PBS buffer (pH: 7.4) were added to the buffer chamber. After incubation in a constant-temperature shaking incubator for 6 h (37 °C), samples from the sample chamber and buffer chamber were diluted 1:1 with PBS or matrix, respectively, and proteins were precipitated with acetonitrile containing IS.

### 4.7. CYP Phenotyping of MT

Recombinant enzyme method [40]: Working solution containing MT was added to humanized recombinant enzymes expressing different subtypes (rCYP1A2, 2A6, 3A4, 3A5, 2B6, 2C8, 2C9, 2C19, 2D6, 2E1, 2J2, 4F2) and pre-incubated at 37 °C for 5 min, followed by the addition of pre-incubated NADPH to initiate the reaction. The incubation system contained 100 mM PBS, 5 mM MgCl_2_, rCYP at a final concentration of 20 pmol/mL, 1 mM NADPH, and 1 μM MT (with DMSO content < 0.1% and organic solvent content < 1% in the system) [41]. At 0, 5, 15, 30, and 60 min, 20 μL of the incubation solution were withdrawn and added to 180 μL of ice-cold acetonitrile (containing 10 ng/mL MT-D4 as internal standard) to terminate the reaction. All samples were prepared in triplicate, with simultaneous setup of negative control groups (empty vector) and positive control groups. The positive substrates included: phenacetin, coumarin, midazolam, testosterone, bupropion, amodiaquine, diclofenac, S-mephenytoin, dextromethorphan, chlorzoxazone, astemizole, and lauric acid.

Chemical inhibitor method [42,43]: Based on the results of the recombinant enzyme assay, positive inhibitors of CYP1A2 and CYP2C19 were used for validation. Working solutions of MT and α-naphthoflavone (CYP1A2 inhibitor) or nootkatone (CYP2C19 inhibitor) were added to the human liver microsome solution and pre-incubated at 37 °C for 5 min, followed by the addition of pre-incubated NADPH to initiate the reaction. The incubation system contained 100 mM PBS, 5 mM MgCl_2_, liver microsomes at 0.5 mg/mL, 1 mM NADPH, 1 μM MT, and 1 μM α-naphthoflavone or 300 μM nootkatone (with DMSO content < 0.1% and organic solvent content < 1%). At 0, 5, 15, and 30 min of incubation, 20 μL of the incubation mixture were withdrawn and added to 180 μL of ice-cold acetonitrile (containing 10 ng/mL MT-D4) to terminate the reaction. All samples were prepared in triplicate, with simultaneous setup of negative controls (without NADPH) and positive control groups.

### 4.8. Enzymatic Kinetics Study on 6-Hydroxylation of Melatonin

Serial concentrations of MT working solutions were added to human or rat liver microsome working solutions and pre-incubated at 37 °C for 5 min, followed by the addition of co-preincubated NADPH working solution. The final concentrations of MT in the incubation system were 8, 16, 32, 64, 128, 256, 512, and 1024 μM, with a final NADPH concentration of 1 mM and a liver microsomal protein concentration of 0.5 mg/mL. After 15 min of incubation, 20 μL aliquots of the incubation mixture were transferred to 180 μL of ice-cold acetonitrile (containing internal standard MT-D4) to terminate the reaction. The content of 6-HMT was determined by LC-MS/MS.

### 4.9. Investigation on the In Vitro Metabolic Effect of MT on CBD

Liver microsomes (human, rat, and beagle) containing working solutions of MT and CBD were pre-incubated at 37 °C for 5 min. The reaction was initiated by adding pre-incubated NADPH. The system contained liver microsomes at 0.5 mg/mL and NADPH at 1 mM (both diluted in PBS containing MgCl_2_). For human liver microsomes, MT concentrations were 0, 0.25, 0.5, 1, 2, 4, 8, 16, and 32 μM; for rat and beagle liver microsomes, MT concentrations were 0, 0.5, 1, 2, 4, 8, 16, and 32 μM (DMSO content < 0.1%, organic solvent content < 1%). The concentration of CBD was 1 μM. After incubation (5 min for rat liver microsomes, 15 min for human and beagle liver microsomes), 20 μL of the incubation mixture were added to 180 μL of ice-cold acetonitrile (containing 10 ng/mL MT-D4) to terminate the reaction. All samples were prepared in triplicate. The content of CBD was determined by LC-MS/MS.

### 4.10. Competitive Inhibition of CBD on MT 6-Hydroxylation

Liver microsomes (human, rat, and beagle) containing CBD and MT at various concentrations were pre-incubated at 37 °C for 5 min, followed by the addition of pre-incubated NADPH to initiate the reaction. The final incubation system contained 100 mM PBS, 5 mM MgCl_2_, 0.5 mg/mL liver microsomal protein, and 1 mM NADPH. The concentrations of CBD in rat and human liver microsomes were 0, 0.25, 0.5, 1, 2, 4, 8, 16, and 32 μM, respectively, while those in beagle liver microsomes were 0, 0.25, 0.5, 1, 2, 4, 8, 16, 32, and 64 μM (with DMSO content < 0.1% and total organic solvent content < 1% in the system). The final concentration of MT was 1 μM. After 30 min of incubation for all species, 20 μL of each sample were mixed with 180 μL of ice-cold acetonitrile (containing 10 ng/mL MT-D4) to terminate the reaction. All samples were prepared in triplicate, and the content of 6-HMT was determined by LC-MS/MS.

### 4.11. Time-Dependent Inhibition of CBD on MT 6-Hydroxylation Using the IC_50_ Shift Method

The time-dependent inhibitory effect of CBD on the 6-hydroxylation of MT was investigated using the IC_50_ shift method [44,45]. Different concentrations of CBD were pre-incubated with human, rat, or beagle dog liver microsomes at 37 °C for 5 min. The reaction was initiated by adding pre-warmed NADPH (control groups lacked NADPH). The final incubation mixture contained 100 mM PBS, 5 mM MgCl_2_, liver microsomes (0.5 mg/mL), 1 mM NADPH, and 0.2% BSA. The CBD concentrations in rat and human liver microsomal incubations were 0, 0.25, 0.5, 1, 2, 4, 8, 16, and 32 μM. Concentrations in beagle dog microsomal incubations were 0, 0.125, 0.25, 0.5, 1, 2, 4, 8, 16, and 32 μM. After a 30 min incubation, the MT solution was added to achieve a final concentration of 2 μM. After a further 30 min incubation, 20 μL of aliquots were removed and added to 180 μL of ice-cold acetonitrile (containing 10 ng/mL MT-D4 as internal standard) to terminate the reaction by vortexing for 1 min. All samples were prepared in triplicate. The concentration of 6-HMT was quantified using LC-MS/MS.

### 4.12. Mechanism-Based Inhibition of CBD on MT 6-Hydroxylation

Different concentrations of CBD were added to human and rat liver microsome incubation solutions, followed by pre-incubation at 37 °C for 5 min. The reaction was initiated by adding the NADPH working solution that had also been pre-incubated for 5 min. The primary incubation system contained 100 mM PBS, 5 mM MgCl_2_, 1 mg/mL liver microsomal protein, 1 mM NADPH, 0.2% BSA, and CBD at final concentrations of 0, 10, 20, 40, 80, 160, and 320 μM (with DMSO content < 0.1% and total organic solvent content < 1% in the system). After pre-incubation for different durations (0, 5, 10, 20, 30 min), 10 μL of the incubation mixture were transferred to a secondary incubation system containing 148 μL PBS and 2 μL MT working solution (final MT concentration: 200 μM). An additional 40 μL of NADPH were added, and the mixture was incubated for 30 min. Subsequently, 20 μL of the sample were mixed with 180 μL of ice-cold acetonitrile (containing 10 ng/mL MT-D4) and vortexed for 1 min to terminate the reaction. All samples were prepared in triplicate, and the content of 6-HMT was determined by LC-MS/MS.

### 4.13. Project the Magnitude of CBD on MT In Vivo Exposure Based on Static Model

The inhibition intensity of CBD on the in vivo metabolism of MT, expressed as AUCR, is defined as the ratio of AUC[I] (AUC when co-administered with the inhibitor) to AUC[contr] (AUC without the inhibitor). A mathematical model describing the mixed inhibition (competitive and irreversible inhibition) of hepatic metabolic enzymes was used to predict the potential for DDI between CBD and MT. AUCR was calculated according to the following formula [45]:

(1) $$C_{\mathrm{inlet},\;\max,\;u}=f_{u,p}\times \left(C\max+\frac{F_{a}\times F_{g}\times K_{a}\times \mathrm{Dose}}{R_{b/p}\times Q_{h}}\right)$$

(2) $$\mathrm{UCR}=\frac{\mathrm{AUC}\left[I\right]}{\mathrm{AUC}\left[\mathrm{contr}\right]}=\frac{1}{\left(\left(\frac{1}{1+\frac{\left[I\right]h}{K_{i}}}\times \frac{1}{1+\left(\frac{K_{\mathrm{inact}}\times \left[I\right]h}{\left(K_{I}+\left[I\right]h\right)\times K_{\deg,\mathrm{CYPX},h}}\right)}\right)\times f_{m}\left({\mathrm{CYP}}_{X}\right)\right)+(1-f_{m}\left({\mathrm{CYP}}_{X}\right))}$$

Wherein, in Formula (1), C_inlet,max,u_ represents the calculation method for the free concentration of CBD at the liver inlet after oral administration of CBD; the calculation result is substituted into [I]h in Formula (2). For human data [30], the value of 3.55 μM after oral administration of 2000 mg CBD reported in the literature was adopted. K_a_ is the oral absorption rate of CBD; it is assumed that the absorption rate is equal to the elimination rate at t_max_, and 90% of the absorption is completed after five absorption half-lives, so K_a_ is 0.693/(t_max_/5) [46]. F_a_ is the absorption fraction of CBD after oral administration, and F_g_ is the fraction of CBD escaping first-pass intestinal metabolism. In Formula (2), K_deg,CYPX,h_ represents the degradation rate of CYP1A2 in the liver, with the value of 0.018/h reported in the literature [28,47]. f_m(CYP1A2)_ is the contribution fraction of CYP1A2-mediated metabolism of MT. f_u,p_ is the free fraction of CBD in plasma, R_b/p_ is the whole blood-to-plasma partition ratio, and Q_h_ is the hepatic blood flow. All parameters used for DDI prediction are listed in Table 6.

To exclude interference from PEPT1 during the absorption phase and more accurately predict the role of CYP enzymes, the observed AUCR values for MT exposure during the elimination phase in animals were calculated using dog AUCR_(0.75h-t)_ and the rat AUCR_(1h-t)_. These values were used to validate the model, and finally, the intensity of DDI induced by CBD on MT in humans was predicted.

### 4.14. LC-MS/MS Methodology for the Quantitation of CBD, MT, and 6-HMT

In this research, samples were obtained from in vivo blood collections of different species, in vitro cell experiments, and incubation experiments. The steps for processing different matrix samples are as follows: extract 20 μL of the sample and then incorporate 180 μL of acetonitrile solution containing MT-D4 at a concentration of 10 ng/mL. Subsequently, the resultant mixture was vortexed for 1 minute and centrifuged at 4 °C for 10 min at 18,800× *g*. The supernatant was then collected for further LC-MS/MS analysis to determine the concentrations of CBD, MT, and 6-HMT [38]. The calibration curves for CBD, MT, and 6-HMT were linear over the concentration range of 2 to 1000 ng/mL. The lowest limit of quantification (LLOQ) was established at 2 ng/mL. All calibration curves exhibited excellent linearity with correlation coefficients (r^2^) ≥ 0.995, meeting the bioanalytical validation requirements. Methodological validation data are provided in Supplementary Materials Figures S1 and S2 and Tables S1–S6.

Determination of CBD, MT, and 6-HMT in biological samples by high-performance liquid chromatography (HPLC-30AD) was coupled with a tandem triple quadrupole mass spectrometer (Shimadzu 8060). Kromasil 100-5-C8 (2.1 × 50 mm) was selected for the separation of samples. The mobile phase consisted of solvent A (water with 0.1% formic acid and 5 mM ammonium acetate) and solvent B (methanol with 0.1% formic acid). For CBD, MT, and 6-HMT, chromatographic separation was achieved on a Kromasil 100-5-C8 (2.1 × 50 mm). The column temperature was set at 40 °C. The two eluents were 0.1% formic acid and 5 mM ammonium acetate in water (A) and 0.1% formic acid in methanol (B). The mobile phase was delivered at a flow rate of 0.5 mL/min using a gradient of A and B as follows: 0.0–0.5 min: 5% B; 0.5–1.5 min: 5–95% B; 1.5–3.0 min: 95% B; 3.0–3.1 min: 95–5% B; 3.1–4.0 min: 5% B. CBD, MT, 6-HMT, and internal standard MT-D4 were detected by positive electrospray ionization (ESI) ion source with multiple reaction monitoring (MRM) transitions of *m*/*z* 315.15 to 193.10, *m*/*z* 233.15 to 174.10, *m*/*z* 249.15 to 158.14, and *m*/*z* 237.20 to 178.20, respectively. The main mass spectrometry detection parameters were: dry gas 10 L/min, heated gas 10 L/min, collision gas 270 kPa, ion source temperature 300 °C, desolvation temperature 250 °C, and heating module temperature 400 °C.

### 4.15. Data Analysis

Pharmacokinetic parameters: GraphPad Prism 10.1 software was utilized to draw the curves. Noncompartmental analysis was selected to calculate the major PK parameters, including clearance (CL), volume (V), half-life (t_1/2_), peak concentration (C_max_), and area under the curve (AUC), using WinNonlin 8.1. Bioavailability (F) was calculated following Formula (3).

(3) $$F\;=\frac{{\mathrm{AUC}}_{\left(0-\infty ,\;\mathrm{extravascular}\right)}\;\times \;{\mathrm{Dose}}_{i.v.}}{{\mathrm{AUC}}_{\left(0-\infty ,\;i.v.\right)}\;\times \;{\mathrm{Dose}}_{\mathrm{extravascular}}}\times 100\%$$

SPSS 29.0.2.0 software was used to perform unpaired t-tests on the pharmacokinetic parameters (including C_max_ and AUC) of MT in the MT alone group and the MT combined with CBD group, aiming to analyze the effect of CBD on the in vivo exposure of MT and 6-HMT. Similarly, the effect of MT on the pharmacokinetics of CBD was statistically analyzed using the same method. A *p*-value < 0.05 was considered statistically significant, and *p* < 0.01 was regarded as statistically highly significant.

Cell uptake assessment: The concentration of the analyte in cells was corrected using the protein concentration of the cell lysate in each well.

(4) $${Conc}_{lysate}=\frac{{\mathrm{Conc}}_{\mathrm{drug}}}{{\mathrm{Conc}}_{\mathrm{Protein}}}$$

The uptake rate (U, pmol/mg/min) of the compound was calculated following Formula (5).

(5) $$U=\frac{C_{\mathrm{lysate}}}{P\times t}$$

where C_lysate_ is the amount of drug in the cell lysate (ng); P is cellular protein content (mg); t is incubation time (min).

The uptake ratio of the compound was calculated following Formula (6)

(6) $$\mathrm{UR}=\frac{U}{\;U_{\mathrm{MOCK}}}$$

where U_MOCK_ is the uptake rate in MOCK-MDCK cells (pmol/mg/min).

The inhibition ratio was calculated following Formula (7)

(7) $$\mathrm{IR}=(1-\frac{U_{\mathrm{with}\;\mathrm{inhibitor}}-U_{\mathrm{mock}\;\mathrm{with}\;\mathrm{inhibitor}}}{U_{\mathrm{without}\;\mathrm{inhibitor}}-U_{\mathrm{mock}\;\mathrm{without}\;\mathrm{inhibitor}}})\times 100\%$$

According to the ICH M12 Drug Interaction Studies, when the uptake ratio (UR) is ≥2 and the inhibition ratio by a selective inhibitor is ≥50%, it indicates that the compound is a substrate of the transporter.

Enzymatic kinetic parameters: The enzymatic kinetic parameters (K_m_ and V_max_) of MT in rat or human liver microsomes were calculated using the Enzyme-Menten module of Graphpad Prism 10.1 software.

Competitive inhibition and time-dependent inhibition assays: The production of 6-HMT without inhibitors was set as 100%. The 6-HMT production at different CBD concentrations was compared with that in the zero-concentration group. With the residual CYP enzyme activity as the vertical axis and the logarithm of CBD concentration as the horizontal axis, GraphPad Prism 10.1 was used to plot the 6-HMT production rate curves for different CBD concentration groups, and the IC_50_ values were calculated.

Irreversible inhibition assays: A semi-logarithmic curve was generated by plotting residual enzyme activity against pre-incubation time. The observed CYP enzyme inactivation rate (K_obs_) was derived from the initial slope of the linear regression. The Lineweaver-Burk double reciprocal curve (1/K_obs_ vs. 1/I) described by Formula (8) was used to determine K_I_ and K_inact_: K_inact_ was estimated from the reciprocal of the Y-intercept, and K_I_ was obtained from the negative reciprocal of the X-intercept.

(8) $$\frac{1}{k}=\frac{K_{I}}{K_{\mathrm{inact}}}\frac{1}{\left[I\right]}+\frac{1}{K_{\mathrm{inact}}}$$

## 5. Conclusions

This study identifies for the first time that CBD modulates MT disposition via a dual mechanism, inhibiting PEPT1-mediated intestinal absorption and CYP1A2-dependent metabolic degradation, while MT exerts negligible effects on CBD pharmacokinetics. CBD-induced alterations in MT pharmacokinetics are species-specific: reducing total exposure but stabilizing plasma concentrations in rats, and significantly enhancing MT bioavailability in beagles, attributed to interspecies differences in CYP1A2 inhibition potency and intestinal PEPT1 function. A static mechanistic model-based IVIVE predicts that CBD co-administration could increase MT exposure in humans by 12.48-fold, a clinically relevant magnitude highlighting the need for dosage adjustments in potential combination therapy. These findings fill critical gaps in understanding CBD-MT DDI and provide a pharmacokinetic basis for optimizing CBD-MT formulations, addressing MT’s rapid elimination limitation to improve clinical efficacy and safety.

## Figures and Tables

**Table 1:** Pharmacokinetic parameters of MT, 6-HMT, and CBD after beagles orally administered with MT or MT with CBD ($\bar{x}$(s), *n* = 6).

Parameters	Unit	MT		6-HMT		CBD
		MT + Vehicle 3 mg/kg	MT + CBD (3 + 3) mg/kg	MT + Vehicle 3 mg/kg	MT + CBD (3 + 3) mg/kg	MT + CBD (3 + 3) mg/kg
t_1/2_	h	0.33 (0.04)	0.92 (0.15) **	3.33 (1.11)	4.11 (1.69)	3.25 (1.59)
T_max_	h	0.22 (0.07)	0.71 (0.29) **	0.29 (0.10)	0.58 (0.20)	0.92 (0.13)
C_max_	ng/mL	1082 (356.7)	1355 (239.6)	74.80 (10.94)	62.9 (18.18)	412.5 (172.1)
AUC_(0–t)_	h × ng/mL	601.9 (219.1)	2536 (458.7) **	179.7 (36.31)	192.4 (72.83)	1004 (306.8)
AUC_(0–∞)_		611.7 (222.6)	2547 (461.4) **	202.1 (46.02)	245.7 (69.0)	1063 (336.3)
MRT_(0–t)_	h	0.50 (0.07)	1.66 (0.19) **	3.60 (0.81)	2.94 (0.83)	2.73 (0.19)
AUC_(0–t, 6-HMT/MT)_	/	/	/	0.32 (0.18)	0.07 (0.02) **	/

/: Not available. **: *p* < 0.01, statistically significant difference, compared to 3 mg/kg MT + Vehicle group.

**Table 2:** Pharmacokinetic parameters of MT and 6-HMT in rats after administration of MT with or without CBD via different routes ($\bar{x}$(s), *n* = 6).

Parameters	Unit	MT				6-HMT			
		*i.v.* (mg/kg)	*p.o.* (mg/kg)			*i.v.* (mg/kg)	*p.o.* (mg/kg)		
		MT + CBD	MT + Vehicle	MT + CBD	MT + CBD	MT + CBD	MT + Vehicle	MT + CBD	MT + CBD
		(1 + 1)	5	(5 + 5)	(5 + 20)	(1 + 1)	5	(5 + 5)	(5 + 20)
t_1/2_	h	0.21 (0.05)	0.66 (0.41)	0.66 (0.14)	1.80 (0.94) *	0.28 (0.04)	1.56 (0.90)	1.72 (1.48)	3.41 (1.90)
T_max_		/	0.17 (0.09)	0.33 (0.13)	0.14 (0.09)	/	0.25 (0)	0.33 (0.13)	0.33 (0.20)
C_max_	ng/mL	/	588.3 (371.2)	212.5 (118.9) *	99.05 (36.63) **	/	145.6 (40.82)	125.1 (33.41)	64.17 (10.40) **
AUC_(0–1h)_	h × ng/mL	/	240.6 (153.9)	114 (67.62)	65.25 (19.06) *	/	90.04 (26.46)	77.02 (26.81)	48.98 (8.27) **
AUC_(1h–t)_		/	53.14 (16.16)	39.93 (18.94)	77.67 (29.54) **	/	57.24 (3.24)	50.88 (21.55)	92.28 (16.37) **
AUC_(0–t)_		260.3 (34.63)	267.2 (152.6)	138.1 (76.76)	121.4 (38.04) *	33.63 (5.10)	125.2 (21.64)	110.2 (39.21)	123.2 (15.77)
AUC_(0–t, 6-HMT/MT)_	/	/	/	/	/	0.13 (0.02)	0.56 (0.22)	0.93 (0.44)	1.07 (0.28)
MRT_(0–t)_	h	0.17 (0.03)	0.67 (0.30)	0.65 (0.11)	1.33 (0.31) **	0.38 (0.06)	1.41 (0.59)	0.88 (0.11)	2.37 (0.38) **
V_z_	L/kg	1.16 (0.18)	/	/	/	/	/	/	/
CL	L/h/kg	3.88 (0.45)	/	/	/	/	/	/	/

*: *p* < 0.05, there was a statistical difference, **: *p* < 0.01, there was a significant statistical difference, compared with the 5 mg/kg MT + Vehicle group.

**Table 3:** Pharmacokinetic parameters of CBD after oral co-administration of different doses of MT in rats ($\bar{x}$(s), *n* = 6).

Parameters	Unit	MT + CBD	Vehicle + CBD	MT + CBD
		(5 + 20)	20	(20 + 20)
t_1/2_	h	4.14 (1.30)	3.55 (1.01)	2.67 (0.89)
T_max_		1.50 (0.32)	0.92 (0.20)	1.21 (0.62)
C_max_	ng/mL	509.5 (170.8)	704.5 (514.9)	519.4 (228.6)
AUC_(0–t)_	h × ng/mL	1590 (389.1)	1573 (684.2)	1394 (921.7)
AUC_(0–∞)_		1606 (395.0)	1592 (688.5)	1409 (928.9)
MRT_(0–t)_	h	3.85 (0.54)	3.11 (0.85)	2.71 (0.62)

**Table 4:** Pharmacokinetic parameters of MT and CBD after pre-administration of ABT and co-administration of MT and CBD in rats ($\bar{x}$(s), *n* = 6).

Parameters	Unit	MT				CBD	
		ABT + MT	MT + Vehicle	ABT + MT + CBD	MT + CBD	ABT + MT + CBD	MT + CBD
		5 mg/kg	5 mg/kg	(5 + 20) mg/kg	(5 + 20) mg/kg	(5 + 20) mg/kg	(5 + 20) mg/kg
t_1/2_	h	1.31 (0.48)	0.66 (0.41)	2.99 (3.49)	1.80 (0.94) *	14.36 (18.79)	4.14 (1.30)
T_max_		0.50 (0.47)	0.17 (0.09)	1.68 (0.78)	0.14 (0.09)	1.67 (0.52)	1.50 (0.32)
C_max_	ng/mL	2097 (614.8)	588.3 (371.2)	903.1 (432.9) ^##^	99.05 (36.63) **	514.4 (507.5)	509.5 (170.9)
AUC_(0–t)_	h × ng/mL	8845 (3485)	267.2 (152.6)	3859 (1678) ^#^	121.4 (38.04) *	2691 (2127)	1590 (389.1)
MRT_(0–t)_	h	39.65 (16.10)	0.67 (0.30)	21.70 (13.36)	1.33 (0.31) **	4.78 (1.00)	3.85 (0.54)

**: *p* < 0.01, with significant statistical differences, *: *p* < 0.05, with statistical differences, both of which were compared with the MT 5 mg/kg group. ^##^: *p* < 0.01, with significant statistical differences, ^#^: *p* < 0.05, with statistical differences, both of which were compared with the MT 5 mg/kg group of rats in combination with the ABT model.

**Table 5:** Whole blood/plasma distribution ratios and protein binding rates of CBD and MT in different species ($\bar{x}$ ± s, *n* = 3).

Parameters	Rat		Mouse		Dog		Human	
	CBD	MT	CBD	MT	CBD	MT	CBD	MT
R_b/p_ (Ratio)	0.63 (0.05)	1.11 (0.19)	0.82 (0.19)	0.92 (0.09)	1.07 (0.14)	1.01 (0.02)	0.52 (0.08)	1.08 (0.02)
Plasma Protein Binding (%)	99.75 (0.01)	57.52 (1.52)	99.71 (0.01)	54.92 (1.37)	99.67 (0.06)	63.58 (0.60)	99.90 (0.01)	61.16 (1.59)
Liver Microsome Protein Binding (%)	99.45 (0.01)	2.79 (2.97)	/	/	99.82 (0.01)	21.96 (4.40)	99.27 (0.02)	2.06 (2.91)
Brain Tissue Protein Binding (%)	99.94 (0.01)	68.54 (0.94)	/	/	/	/	/	/

**Table 6:** Parameters of CBD for static DDI prediction models.

Parameters	Beagle	Rat	Human
f_u, p_ (%)	0.33	0.25	0.01
f_u, mic_ (%)	0.18	0.55	0.73
C_max_ (μM)	1.31	1.62	/
Dose (mg)	30	5	2000
F_a_ × F_g_	1	1	1
t_max_ (h)	0.92	1.5	/
K_a_	3.77	2.31	/
R_b/p_	1.07	0.63	0.52
Q_h_ (mL/min)	309	13.8	1450
K_deg CYP1A2_ (/h)	0.018	0.018	0.018
[I]_h_ (nM)	1.37	0.74	3550
IC_50_ (μM)	0.65	9.51	3.39
K_i, u_ (nM)	0.06	2.62	1.24
K_inact_ (/min)	/	/	0.063
K_I_ (μM)	/	/	25.63
K_I, u_ (nM)	/	/	18.71
f_m(CYP1A2)_	0.92	0.92	0.92

f_u,p_: free fraction of CBD in plasma; f_u,mic_: free fraction of CBD in liver microsomes; Dose: dosage of CBD; F_a_ × F_g_: F_a_ represnts the orl absorption fraction, F_g_ represnts the fraction of CBD escaping first pass intestinal metabolism, F_a_ × F_g_ is assumed to 1; K_a_: oral absorption rate of CBD, calculated as 0.693/(t_max_/5); R_b/p_: Blood-to plasma ratio of CBD; Q_h_: hepatic blood flow; K_deg CYP1A2_: the natural degradation rate of hepatic CYP1A2; [I]h: estimated free concentration of CDB in liver; K_i, u_: Assuming competitive inhibition, Ki is estimated as IC_50_/2 and corrected by f_u,mic_ to obtain K_i,u_. K_inact_ represents the maximum inactivation rate; K_I_ is the inhibitor concentration at half of Kinact; K_I,u_ is obtained by correcting KI with fu, mic. f_m(CYP1A2)_ refers to the proportion of MT metabolized by CYP1A2.

## Data Availability

The original contributions presented in this study are included in the article/Supplementary Material. Further inquiries can be directed to the corresponding author.

## Supplementary Materials

The following supporting information can be downloaded at: https://www.mdpi.com/article/10.3390/ph19010080/s1, Figure S1. Representative chromatograms of CBD, MT, 6-HMT and MT-D4 in rat plasma. (a) Rat blank plasma. (b) Rat blank plasma spiked with a mixture of CBD + MT + 6-HMT at LLOQ concentration (2 ng/mL). (c) Actual plasma sample from rats following oral administration. CBD: cannabidiol; MT: melatonin; MT-D4: melatonin-D4. Figure S2. Representative chromatograms of CBD, MT, 6-HMT and MT-D4 in beagle plasma. (a) Beagle blank plasma. (b) Beagle blank plasma spiked with a mixture of CBD + MT + 6-HMT at LLOQ concentration (2 ng/mL). (c) Actual plasma sample from beagles following oral administration. CBD: cannabidiol; MT: melatonin; MT-D4: melatonin-D4. Table S1. Accuracy and precision of LC-MS/MS methods for CBD, MT and 6-HMT in rat plasma. Table S2. Accuracy and precision of LC-MS/MS methods for CBD, MT and 6-HMT in beagle plasma. Table S3. Method recoveries and matrix effects of CBD, MT, and 6-HMT in rat and beagle plasma ($\bar{x}$ ± s, *n* = 6). Table S4. Stability of CBD, MT and 6-HMT in rat plasma (*n* = 3). Table S5. Stability of CBD, MT and 6-HMT in beagle plasma (*n* = 3). Table S6. Dilution accuracy of CBD, MT, and 6-HMT in rat and beagle plasma ($\bar{x}$ ± s, *n* = 6).

## Abbreviations

ABT, 1-Aminobenzotriazole; ADME, Absorption, distribution, metabolism, and excretion; AUC, Area under the plasma concentration versus time curve; AUCR, Ratio of AUC of object drug in the presence to absence of inhibitor; BCA, Bicinchoninic acid; BSA, Albumin from bovine serum; C_max_, peak concentration; CBD, Cannabidiol; CBT-I, cognitive behavioral therapy for insomnia; CEPH, Cefalexin; CL, Clearance; CYP, Cytochrome P450 proteins; DMSO, Dimethyl sulfoxide; DMEM, Dulbecco’s Modified Eagle Medium; FBS, Fetal bovine serum; Gly, Glycyl-sarcosine; HBSS, Hank’s Balanced Salt Solution; IC_50_, 50% Inhibitory concentration; IS, Internal standard; K_m_, Michaelis-Menten constant; K_I_, Inhibition constant; K_inact_, Maximal inactivation rate; LC-MS/MS, Liquid chromatography-tandem mass spectrometry; MES, 2-(N-Morpholino)ethanesulfonic acid; MT, Melatonin; MT-D4, Melatonin-D4; MDCK, Madin-Darby Canine Kidney cells; MOCK, Non-transfected cells; NADPH, Nicotinamide adenine dinucleotide phosphate; PBS, Phosphate Buffered Saline; PEPT, Peptide Transporters; PK, Pharmacokinetic; RED, Rapid equilibrium dialysis; t_1/2_, half-live; TDI, Time-dependent inhibition; UBEN, Ubenimex; V, volume; V_max_, Maximum reaction velocity; 6-HMT, 6-Hydroxymelatonin.
