# Supplementary material for: Unraveling Cannabidiol’s Bidirectional Regulation of Melatonin Pharmacokinetics via PEPT1/CYP1A2: Mechanistic Insights and Quantitative Projections
Source: Pharmaceuticals (Basel). 2025 Dec 30;19(1):80. doi: 10.3390/ph19010080 (PMC12845078; doi:10.3390/ph19010080)
Supplement: Supplementary file 1 [file pharmaceuticals-19-00080-s001.zip › pharmaceuticals-4012704-supplementary.pdf]

Supplementary Material:

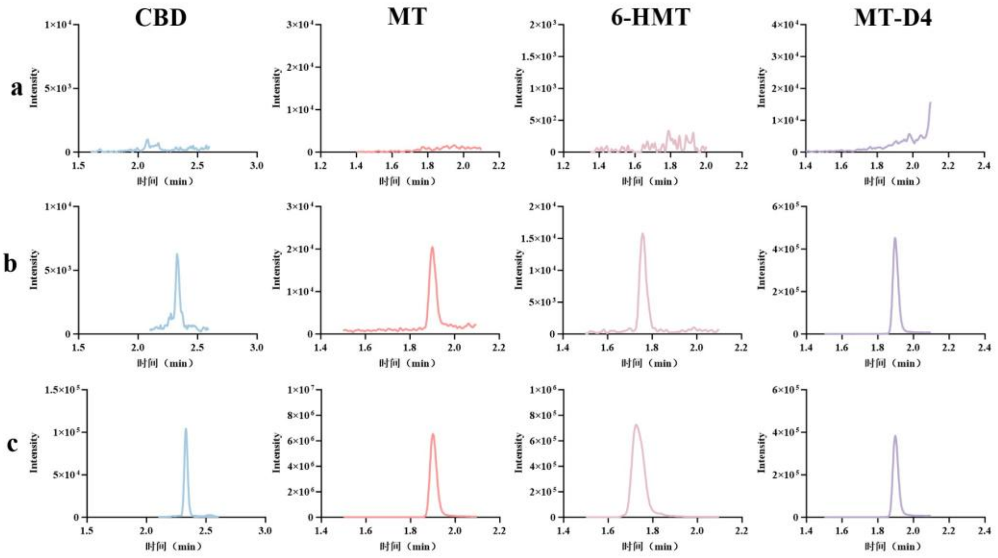

Figure S1. Representative chromatograms of CBD, MT, 6-HMT and MT-D4 in rat plasma. (a) Rat blank plasma. (b) Rat blank plasma spiked with a mixture of CBD + MT + 6-HMT at LLOQ concentration (2 ng/mL). (c) Actual plasma sample from rats following oral administration. CBD: cannabidiol; MT: melatonin; MT-D4: melatonin-D4.

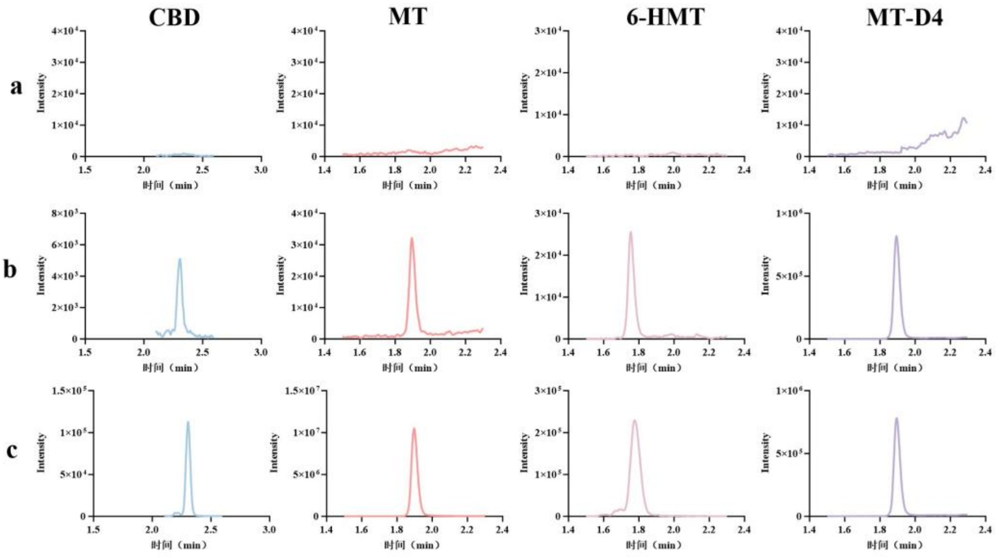

Figure S2. Representative chromatograms of CBD, MT, 6-HMT and MT-D4 in beagle plasma. (a) Beagle blank plasma. (b) Beagle blank plasma spiked with a mixture of CBD + MT + 6-HMT at LLOQ concentration (2 ng/mL). (c) Actual plasma sample from beagles following oral administration. CBD: cannabidiol; MT: melatonin; MT-D4: melatonin-D4.

Table S1. Accuracy and precision of LC-MS/MS methods for CBD, MT and 6-HMT in rat plasma.

Analytes	Concentration (ng/mL)	Intra-Day (*n*=6)						Inter-Day (*n*=18)	
		RE (%)			RSD (%)			RE (%)	RSD (%)
CBD	2	14.35	8.71	8.34	2.71	6.15	6.94	14.47	5.77
	4	1.32	-3.05	-3.45	1.84	4.22	2.77	4.85	3.64
	80	-2.51	-3.68	-0.81	1.08	1.38	1.03	-3.09	1.66
	800	8.01	8.51	10.55	1.13	1.15	0.82	3.63	1.48
MT	2	1.51	-2.73	5.74	4.45	3.79	7.04	1.51	6.14
	4	-1.46	-4.18	-4.75	3.09	3.23	3.26	-3.46	3.39
	80	-1.55	-3.90	-4.55	1.13	1.35	1.63	-3.39	1.92
	800	2.86	8.56	5.96	1.75	1.21	1.57	5.84	2.64
6-HMT	2	-4.44	-0.32	-0.21	2.62	2.68	2.44	-1.78	3.20
	4	-3.44	-3.18	-7.87	2.19	3.45	1.93	-4.81	3.43
	80	-4.67	-5.44	-2.48	0.94	1.67	0.98	-4.26	1.75
	800	10.05	12.49	13.67	1.81	1.47	1.31	12.03	2.03

Table S2. Accuracy and precision of LC-MS/MS methods for CBD, MT and 6-HMT in beagle plasma.

Analytes	Concentration (ng/mL)	Intra-Day (*n*=6)						Inter-Day (*n*=18)	
		RE (%)			RSD (%)			RE (%)	RSD (%)
CBD	2	7.65	13.72	9.48	8.01	3.23	5.47	10.28	5.96
	4	-3.22	1.43	-7.55	3.83	4.98	5.66	-3.11	6.00
	80	4.44	3.44	-3.06	3.40	3.66	6.50	1.61	5.51
	800	12.74	11.84	10.81	1.74	2.64	4.94	11.80	3.25
MT	2	10.71	-5.76	-4.61	8.04	6.56	5.98	0.11	10.17
	4	7.69	0.81	0.18	2.25	3.23	3.98	2.89	4.53
	80	1.84	1.52	2.35	1.39	0.58	1.19	1.90	1.10
	800	9.82	10.17	12.52	0.76	1.02	0.82	10.84	1.38
6-HMT	2	-3.30	-4.10	-2.38	3.23	4.26	3.84	-3.26	3.64
	4	-3.54	-4.42	-7.81	1.30	3.62	3.39	-5.26	3.41
	80	-2.81	-2.94	-6.04	1.03	0.77	1.65	-3.93	1.95
	800	12.18	13.41	6.00	0.92	0.67	2.76	10.53	3.40

Table S3. Method recoveries and matrix effects of CBD, MT, and 6-HMT in rat and beagle plasma (x̄±s, *n*=6).

Species	Analytes	Concentration (ng/mL)	Extraction Recovery (%)	Matrix Effect (%)	Matrix Effect RSD (%)
Rat	CBD	4	98.71±3.32	102.17±5.40	5.29
		80	97.21±1.42	/	/
		800	94.45±1.13	98.15±0.91	0.93
	MT	4	99.12±1.67	96.46±2.21	2.29
		80	101.04±1.93	/	/
		800	95.52±0.84	96.59±1.23	1.27
	6-HMT	4	103.71±2.17	110.25±4.71	4.27
		80	101.43±1.72	/	/
		800	95.38±1.10	101.93±2.08	2.04
Beagle	CBD	4	95.24±5.42	101.77±2.39	2.35
		80	101.34±5.29	/	/
		800	99.71±7.04	98.87±4.39	4.44
	MT	4	98.15±4.42	106.79±4.83	4.52
		80	96.25±3.87	/	/
		800	94.43±4.50	104.56±5.00	4.78
	6-HMT	4	101.14±2.86	99.60±9.25	9.28
		80	96.52±3.61	/	/
		800	94.19±4.04	102.03±4.32	4.23

Table S4. Stability of CBD, MT and 6-HMT in rat plasma (*n*=3).

Analytes	Concentration (ng/mL)	RT for 4 h		4℃ for 24 h		Freeze–thaw three times		-40℃ for 30 d	
		RE (%)	RSD (%)	RE (%)	RSD (%)	RE (%)	RSD (%)	RE (%)	RSD (%)
CBD	4	-6.91	10.62	-1.04	10.48	-1.34	7.96	-10.20	4.42
	80	2.65	2.36	0.64	1.05	0.97	2.25	-5.92	1.06
	800	-2.34	0.34	-2.40	3.52	1.54	0.42	-4.76	0.64
MT	4	4.96	5.37	7.97	0.65	7.92	3.29	-3.34	3.72
	80	6.02	2.35	2.72	0.83	2.59	1.85	-4.46	0.64
	800	1.33	0.78	-3.16	3.91	1.63	0.53	-5.23	0.41
6-HMT	4	14.44	1.14	14.53	2.84	14.86	0.96	12.91	1.14
	80	10.25	3.26	5.94	1.04	7.14	2.63	-0.26	1.22
	800	1.91	0.23	-2.96	3.91	3.34	0.80	-3.63	0.54

RT: Room temperature.

Table S5. Stability of CBD, MT and 6-HMT in beagle plasma (*n*=3).

Analytes	Concentration (ng/mL)	RT for 4 h		4℃ for 24 h		Freeze–thaw three times		-40℃ for 30 d	
		RE (%)	RSD (%)	RE (%)	RSD (%)	RE (%)	RSD (%)	RE (%)	RSD (%)
CBD	4	3.47	4.81	5.08	2.39	6.20	6.30	-3.93	6.59
	80	-1.28	6.88	-3.01	3.63	1.18	1.09	1.46	8.10
	800	-2.59	4.47	-1.34	2.57	-1.50	1.24	-0.04	7.62
MT	4	0.29	5.32	-0.52	4.36	1.36	6.24	1.95	0.66
	80	5.00	1.63	2.69	1.30	3.18	0.74	0.89	3.66
	800	3.49	1.38	2.18	0.60	-8.08	3.62	1.94	0.94
6-HMT	4	2.23	3.58	-0.67	1.85	5.76	0.52	3.33	2.97
	80	4.97	2.66	1.61	2.07	2.97	1.40	-1.09	6.31
	800	1.38	1.78	-1.42	4.03	-0.95	2.84	6.21	0.41

RT: Room temperature.

Table S6. Dilution accuracy of CBD, MT, and 6-HMT in rat and beagle plasma ($\bar{x}$±s, *n*=6)

Species	Analytes	Concentration (ng/mL)	10-fold diluted (ng/mL)	Accuracy (%)	RSD (%)
Rat	CBD	2000	200	105.15±3.09	2.94
	MT	2000	200	105.99±2.46	2.32
	6-HMT	2000	200	109.91±2.98	2.71
Beagle	CBD	2000	200	95.53±9.24	9.68
	MT	2000	200	99.25±2.23	2.24
	6-HMT	2000	200	102.69±2.49	2.42
